# Differential diagnosis of atypical encephalopathy in critical care: a case report

**DOI:** 10.1186/s12879-020-05492-8

**Published:** 2020-10-16

**Authors:** Chung Li, Ming-Yieh Peng, Chia-Hui Chang, Yuan-Yu Hsu, Min-Shiau Hsieh, Shinn-Kuang Lin, Yi-Hsin Lee, Mei-Chen Yang

**Affiliations:** 1 Department of Internal Medicine, Taipei Tzu Chi Hospital, Buddhist Tzu Chi Medical Foundation, New Taipei, Taiwan; 2 Division of Infection Diseases and Tropical Medicine, Department of Internal Medicine, Taipei Tzu Chi Hospital, Buddhist Tzu Chi Medical Foundation, New Taipei, Taiwan; 3 School of Medicine, Tzu Chi University, Hualien, Taiwan; 4 Division of Endocrine and Metabolism, Department of Internal Medicine, Taipei Tzu Chi Hospital, Buddhist Tzu Chi Medical Foundation, New Taipei, Taiwan; 5 Department of Medical Imaging, Taipei Tzu Chi Hospital, Buddhist Tzu Chi Medical Foundation, New Taipei, Taiwan; 6 Division of Thoracic Surgery, Department of Surgery, Taipei Tzu Chi Hospital, Buddhist Tzu Chi Medical Foundation, New Taipei, Taiwan; 7 Department of Neurology, Taipei Tzu Chi Hospital, Buddhist Tzu Chi Medical Foundation, New Taipei, Taiwan; 8 Department of Anatomy Pathology, Taipei Tzu Chi Hospital, Buddhist Tzu Chi Medical Foundation, New Taipei, Taiwan; 9 Division of Pulmonary Medicine, Department of Internal Medicine, Taipei Tzu Chi Hospital, Buddhist Tzu Chi Medical Foundation, No. 289, Jianguo Rd, Xindian District New Taipei, 23143 Taiwan

**Keywords:** Neurocysticercosis, Encephalopathy, Pulmonary aspergillosis, Pyogenic ventriculitis, *Streptococcus intermedius*, Diabetic ketoacidosis

## Abstract

**Background:**

A lower level of consciousness is a common presentation in critical care, with many different causes and contributory factors, of which more than one may be present concurrently.

**Case presentation:**

We described a woman with poorly controlled diabetes and steroid-dependent asthma who presented in a deep coma. She was found to have *Streptococcus intermedius* bacteremia and pyogenic ventriculitis that originated from right middle lobe pneumonia. Also, multiple small parenchymal lesions were observed on brain magnetic resonance imaging and increased protein concentration was noted in cerebral spinal fluid. Initially, her coma was thought to be due to diabetic ketoacidosis and septic encephalopathy. However, her lowered level of consciousness was disproportionate to either diabetic ketoacidosis or septic encephalopathy, and her clinical course was not as expected for these two conditions. Treatment with antibiotic, corticosteroid and antihelminthic drugs was administered resulting in improving consciousness. The *Streptococcus intermedius* pneumonia progressed to form a large cavity that needed an early surgical lobectomy and resulted in the unexpected diagnosis of chronic cavitary pulmonary aspergillosus.

**Conclusions:**

In critical care, a lowered level of consciousness may have many etiologies, and critical care clinicians should be familiar with the signs and symptoms of all possible causes to enable prompt diagnosis and appropriate treatment.

## Background

A lower level of consciousness is a common presentation in critical care. The most common contributing factors are stroke, post-anoxic coma, poisoning, and metabolic problems [1]. Many unconscious patients in critical conditions have more than one contributing factor on admission. Unconsciousness due to diabetic ketoacidosis (DKA) induced by an infection or stroke is also common in critical care [2]. However, DKA alone occasionally causes a lowered level of consciousness, which usually resolves within 5 days after adequate treatment [3], but it may take up to 6 months to regain cognitive function [4]. Routine neuroimaging examinations for excluding stroke in patients with DKA are not cost-effective, unless patients have atypical neurological signs. Up to 70% of patients with septic encephalopathy experience various degrees of cerebral dysfunction, which generally resolves within a week with adequate antibiotic treatment but may lead to a long-term cognitive impairment [5, 6]. In critical care, patients with a delay in recovery of consciousness of over a week require further evaluation.

Parasitic infections are uncommon in highly developed countries. However, with the advancement of technology, traveling abroad has become common, and patients may develop parasitic infections during visits to endemic areas. This may result in delayed diagnosis and treatment because clinicians from non-endemic areas are not familiar with these conditions. Neurocysticercosis is a medical emergency that requires timely diagnosis and treatment [7]. The diagnosis of neurocysticercosis depends on histological findings from brain biopsy or neuroimaging, with or without an exposure history [8]. However, it is often impossible to obtain an exposure history. Although brain magnetic resonance imaging (MRI) can provide more information and is better for the diagnosis of neurocysticercosis, it is expensive and time-consuming and is often not considered as the initial neuroimaging study in critical care [9].

Here, we reported a woman with poorly controlled diabetes mellitus and chronic steroid-dependent asthma, who presented with unconsciousness due to multiple factors and finally had a satisfactory outcome.

## Case presentation

A 69-year-old woman with poorly controlled diabetes mellitus and steroid-dependent asthma arrived at the Emergency Department in a deep coma. She lived alone but maintained daily contact with her son by phone. On the day of her admission, her son did not receive a response to his phone call and called the police to break into her house.

**Fig. 1:**
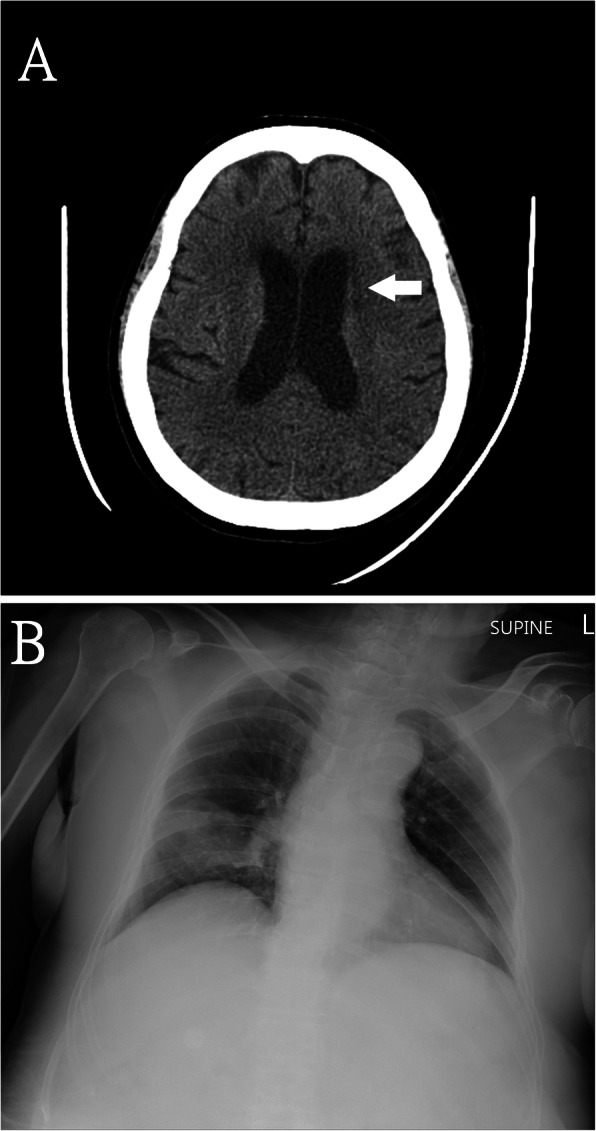
The brain computed tomography and chest radiograph. The brain computed tomography scan revealed a hypodense region in the left corona radiata (Fig. 1a). The chest radiograph revealed right middle lobe pneumonia (Fig. 1b)

**Fig. 2:**
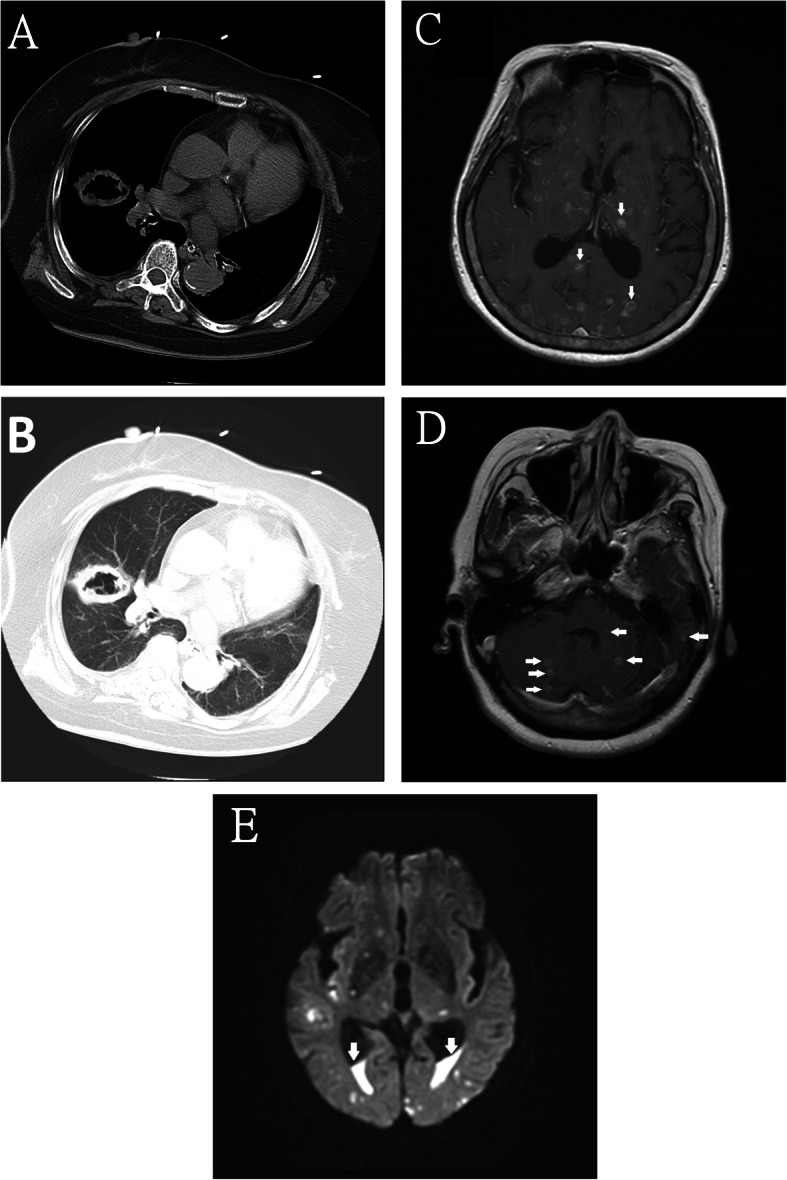
The chest computed tomography and brain magnetic resonance imaging. The chest computed tomography showed a 5.2 cm cavity containing some small ball-like lesions and slight pericardial effusion (Fig. 2a and b). The brain magnetic resonance imaging showed multiple contrast-enhanced cystic lesions with surrounding brain edema and eccentrically hyperdense lesions within the cysts in the cerebrum, cerebellum, and the brain stem (Fig. 2c, d) and abnormal fluid accumulation in the dependent portion of both lateral ventricles (Fig. 2e)

On physical examination, her Glasgow Coma Scale was E1V1M1, blood pressure was 151/87 mmHg, heart rate was 98 beats per minute, respiratory rate was 18 breaths per minute, and body temperature was 37.2 °C. Arterial blood analysis revealed metabolic acidosis and oxygen desaturation (Table 1). Blood biochemistry revealed hyperglycemia with hyperosmolality, ketoacidosis, elevated lactate, and pre-renal azotemia (Table 1). There was leukocytosis with a left shift, indicating the patient had sepsis. Urinary analysis also revealed ketonuria. A brain computed tomography (CT) scan revealed a small hypodense region in the left corona radiata, suggestive of an ischemic stroke (Fig. 1a). The chest X-ray (CXR) revealed right middle lobe pneumonia (Fig. 1b). The electroencephalogram (EEG) revealed diffuse cortical dysfunction but no epileptiform discharges. Kernig’s sign and Brudzinski’s sign were negative. The blood culture 3 days after admission revealed *Streptococcus intermedius* bacteremia which was sensitive to erythromycin, penicillin, vancomycin, linezolid, clindamycin, ceftriaxone, levofloxacin and cefepime. (The minimum inhibitory concentration of penicillin was 0.064 μg/mL) The blood culture showed no fungal growth. Sputum cultures were negative for bacteria, *Mycobacterium tuberculosis*, and fungus (Table 2). She was provided with empiric ceftriaxone on admission, insulin therapy, high-dose hydrocortisone (100 mg every 12 h) for possibly iatrogenic adrenal insufficiency, and fluid replacement. Her pneumonia rapidly progressed, and on Day 4, her chest CT showed a 5.2-cm cavity containing some small ball-like lesions and a slight pericardial effusion (Fig. 2a and b). The hydrocortisone dosage was tapered because of her poorly controlled pneumonia. She remained comatose on Day 7. As her bilaterally symmetrical sensory and motor dysfunctions were not compatible with left corona radiata infarction, and she did not recover her consciousness as expected, brain MRI was performed which showed multiple contrast-enhanced cystic lesions with brain edema and eccentrically located hyperdense lesions within the lesions in the cerebrum, cerebellum, and brain stem (Fig. 2c, d). In addition, abnormal fluid accumulation in the dependent portion of the bilateral lateral ventricle was observed (Fig. 2e), suggesting pyogenic ventriculitis. As the patient was still comatose, we were unable to determine whether she had a history of possible exposure to *Taenia solium*. The stool analysis showed no parasites. Her son recalled that she had traveled to Vietnam 3 months previously, where she may have eaten raw pork dishes. Therefore, a provisional diagnosis was made of *Taenia solium* infection with neurocysticercosis. The family refused a brain biopsy. Lumbar puncture on Day 9 revealed increased protein level; however, the intracranial pressure was not increased (Table 1). The CSF studies were negative for bacteria, tuberculosis, cryptococcus, aspergillosis, viruses, and malignant cells (Table 2). The CSF specimens were sent to another medical center for parasite testing, but the results were negative. Albendazole and praziquantel were administered, after resuming high-dose corticosteroid and intravenous anti-epileptic agents for 3 days to prevent seizures. After 2 days of this therapeutic scheme, her consciousness dramatically improved to E3V5M6 and she disclosed that she had eaten raw pork dishes while in Vietnam and had experienced persistent fever, malaise, and headache during the previous 2 months.

**Table 1:** The body temperature, blood and biochemical reports and the cerebrospinal fluid study reports of the patient during the admission course

	(Normal range)	Day-1	Day-5	Day-9	Day-14	Day-18	Day-24
**Body temperature**	(35–37 °C)	37.2	38.0	37.7	39.0	37.0	36.9
**Blood test**							
**Arterial blood gas**							
PH	7.31–7.41	7.31	7.44	7.42	7.43	7.44	7.45
PaCO2	41–51 mmHg	26.0	37.1	38.2	41.4	32.2	37.1
PaO2	80–100 mmHg	62.3	87.1	153.5	157.6	105.1	142.0
HCO3	22–26 mmol/L	11.3	24.7	24.3	26.9	21.4	25.1
SaO2	95–100%	87.7	96.4	99.4	99.4	98.2	99.3
**Hemoglobin**	12–16 g/dL	13.1	13.4	10.5	11.4	10.5	10.5
**Platelet**	150–400*10^3^/μL	345.0	343.0	276.0	467.0	403.0	342.0
**WBC**	3.5–11*10^3^/μL	22.84	22.88	14.03	16.85	20.35	10.43
**Differential count**							
Band	0–3%	5.0	2.0	0.0	0.0	2.0	0.0
Neutrophil	40–75%	89.0	78.0	93.0	87.0	92.0	73.1
Lymphocyte	20–45%	3.0	14.0	6.0	9.7	4.0	18.6
Monocyte	2–10%	0.0	0.0	1.0	3.2	1.0	7.2
Eosinophil	1–6%	0.0	0.0	0.0	0.0	0.0	0.9
Basophil	0–1%	0.0	0.0	0.0	0.0	0.0	0.2
Metamyelocyte	0%	1.0	0.0	0.0	0.0	0.0	0.0
**Lactate**	0.4–2 mmol/L	2.2					
**Osmolarity**	275–290 mOsm/Kg	346.0					
**Ketone body**	< 0.6 mmol/L	3.9					
**Blood urea nitrogen**	7–18 mg/dL	52.0	47.0	45.0	38.0	42.0	20.0
**Creatinine**	0.55–1.02 mg/dL	3.0	1.4	1.4	1.2	1.2	0.8
**C-reactive protein**	0–0.33 mg/dL	27.60				5.18	0.12
**Cerebrospinal fluid**							
**Total protein**	15–45 mg/dL			137.4		73.0	
**Sugar**	40–70 mg/dL			45.0		45.0	
**Lactate**	0.6–2.2 mmol/L			4.3		2.6	
**Red cell count**	0–5/μL			158.0		45.0	
**White cell count**	0–5/μL			2.0		9.0	
**Differential count**							
Neutrophil	0–2%			50.0		95.0	
Lymphocyte	63–99%			44.0		5.0	
Monocyte	3–37%			6.0		0.0

**Table 2:** Microorganism culture results for the patient during the admission course

		Day-1	Day-9	Day-14	Day-18
**Blood**	Bacterial culture	Streptococcus^a^	(−)	(−)	(−)
	Fungal culture	(−)	(−)	(−)	(−)
**Serum**	Cryptococcus antigen		(−)		
	Aspergillus antigen		(−)		(−)
**Sputum**	Bacterial culture	(−)	(−)	(−)	(−)
	Fungal culture	(−)		(−)	
	MTB culture	(−)			
	MTB-PCR		(−)		
**Bronchoalveolar lavage**	Bacterial culture		Streptococcus^a^		
	Fungal culture		Aspergillus		
	Cryptococcus antigen		(−)		
	Aspergillus antigen		Positive		
	MTB culture		(−)		
	MTB-PCR		(−)		
	PJP-PCR		(−)		
**Cerebrospinal fluid**	Bacterial culture		(−)		(−)
	Fungal culture		(−)		(−)
	Cryptococcus antigen		(−)		(−)
	Aspergillus antigen		(−)		(−)
	MTB culture		(−)		(−)
	MTB-PCR		(−)		(−)
	HSV1 (IgG/IgM)		(−)		(−)
	HSV2 (IgG/IgM)		(−)		(−)
	VZV IgG		(−)		(−)
**Surgical lung specimens**	Fungus culture			Aspergillus	
	MTB culture			(−)	
	MTB-PCR			(−)	

^a^Streptococcus intermedius

**Abbreviation:** *MTB Mycobacterium tuberculosis*, *PCR* Polymerase chain reaction, *PJP* Pneumocystis jiroveci pneumonia, *HSV* Herpes simplex virus, *VZV* Varicella zoster virus

**Fig. 3:**
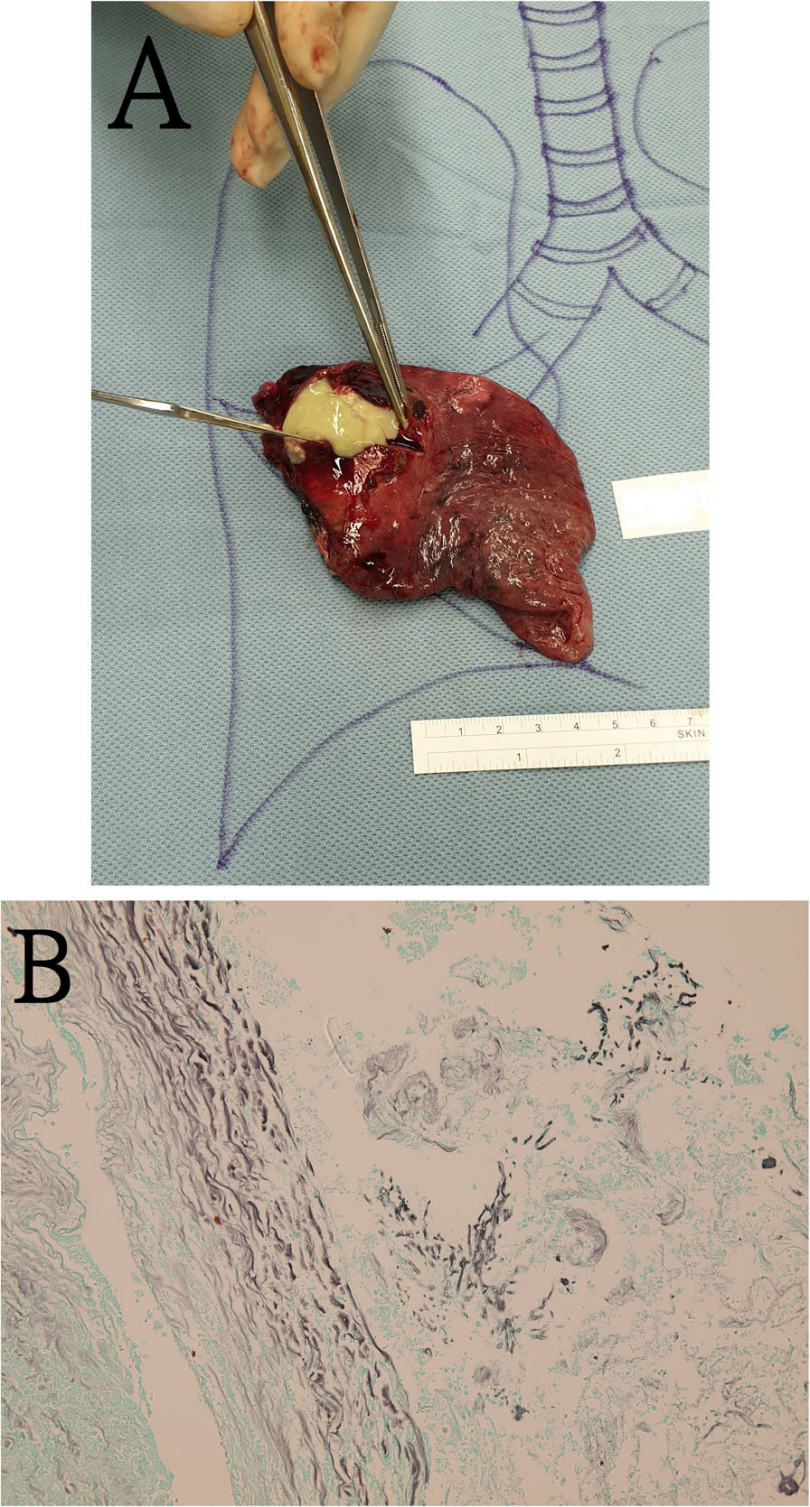
The pathologic findings in the right middle lung. The right middle lobe was surgically removed (Fig. 3a). Histology revealed a chronic abscess cavity, surrounded by a thick fibrous wall and necrotic debris, and focal septate fungal hyphae in the lumen which were positive on Gomori methenamine silver stain (magnification × [200X]) (Fig. 3b)

However, her fever persisted, and we had difficulty with attaining glycemic control. Her right middle lobe cavity was surgically removed on Day 14 to facilitate glycemic control (Fig. 3a). The histology revealed a chronic abscess, surrounded by a thick fibrous wall and some necrotic debris, and focal septate fungal hyphae in the lumen, which were positive on Gomori methenamine silver stain, suggestive of aspergillosis (Fig. 3b). The culture of the surgical lung specimens revealed *Aspergillus* growth. On Day 17, she started a 2-week course of oral voriconazole. On day 17, her consciousness returned to E4V5M6, and a follow-up brain MRI revealed some regression. After 43 days of hospitalization with 1 month of cysticidal therapy, she regained full consciousness, but her lower limb weakness did not improve. Bedside physical therapy for reconditioning rehabilitation to improve muscle endurance and strength, posture training, endurance training and strengthening training were administered for 3 weeks. She was then discharged to a nursing home near her son’s house, where she continued rehabilitation.

## Discussion and conclusions

We have described a 69-year-old woman with poorly controlled diabetes mellitus and steroid-dependent asthma, admitted in a coma due to multiple potential causal factors. The differential diagnosis included disseminated cerebral neurocysticercosis, *Streptococcus intermedius* bacteremia with brain abscesses and pyogenic ventriculitis, and pulmonary aspergillosis. Although the cause of her encephalopathy could not be established with certainty, our patient responded well to combination treatment and made a satisfactory recovery but was left with residual weakness in her legs.

A lowered level of consciousness due to cerebral edema from DKA is rare in adults and generally improves after 5 days although some individuals had residual memory loss and poorer sustained and divided attention at 6 months [4]. Our patient did not improve after 5 days of DKA treatment, suggesting there might be other causes of her coma and lack of consciousness. Therefore, the acute left-sided corona radiata infarction shown in the initial brain CT was considered as a possible cause, but the neurological signs were symmetrical and involved both sensory and motor functions which were not consistent with the diagnosis of a left-sided unilateral corona radiata infarction [10]. Our radiologist thought that the brain MRI showed typical findings of neurocysticercosis which was possibly in the viable phase of disease progression on admission. This could explain why the brain CT did not reveal parenchymal neurocysticercosis or calci. In clinical practice, it is difficult to perform brain MRI on admission for critically non-intubated breathless patients, unless absolutely necessary. Some lesions in our patient had no obvious surrounding brain edema, suggesting possible neurocysticercosis lesions at the beginning of the degenerative phase. This might explain why she did not experience seizures. The magnetic resonance spectroscopy (MRS) for peak lipid measurements may provide more information on how to differentiate a tuberculous brain abscess from neurocysticercosis [11]. However, an MRS was not conducted on this patient for the following reasons: (1) MRS is not routinely ordered in ICU care because it needs patient cooperation. Our patient could not cooperate with an MRS examination. (2) We ordered a brain MRI to assess whether brain infarction was present and we did not expect to find focal lesions suggestive of possible neurocysticercosis. (3) We did not think that MRS imaging was necessary because the radiologist reported that the MRI images provided sufficient evidence of neurocysticercosis. In addition, further MRS images would have taken a further 7 min of examination time; the resolution of the partial volume effect is poor in lesions less than 1 cm^3^ (1 cm × 1 cm × 1 cm) in size; and we were concerned about the patient’s safety.

Although a positive CSF bacterial culture and other signs of bacterial meningitis were absent from our patient, we could not completely rule out the possibility of *Streptococcus intermedius* meningitis since an abnormal fluid accumulation in the dependent portion of the bilateral lateral ventricle typically suggested pyogenic ventriculitis [12]; in addition, concomitant *Streptococcus intermedius* brain and lung abscess had been reported previously [13, 14]. The pyogenic ventriculitis is rare and usually severe, and often causes obvious meningitis signs. The condition commonly comes from hematogenous spreading, trauma or surgical procedures and the most common pathogen implicated is methacillin-resistance *Staphylococcus aureus*. The MRI finding of our patient showed sticky debris or purulent material within the brain lateral ventriculi, which was compatible with pyogenic ventriculitis. The CSF culture for bacteria was negative possibly because the previous antibiotic and corticosteroid use hampered the CSF bacterial culture results, prohibiting the bacterial meningitis sign.

Most patients with septic encephalopathy have marked global brain dysfunction presenting as confusion or coma, but usually fully recovered within a week of onset, if adequately treated [5, 6, 15]. The most sensitive tools for diagnosing septic encephalopathy in critical care are clinical examination and EEG, and neuroimaging is used only for exclusion of other diagnoses [15]. The EEG findings of our patient suggested metabolic encephalopathy, which was compatible with a diagnosis of septic encephalopathy. However, her level of consciousness did not improve after adequate antibiotic treatment for 1 week, but rapidly improved within 2 days after initiating cysticidal drugs and high dose of corticosteroids. Therefore, there is more than one possible cause of the encephalopathy in our patient, including neurocysticercosis and *Streptococcus intermedius* infection. In neurocysticercosis, CSF testing is not necessary but used to rule out other conditions [8]. The CSF testing of our patient did not establish a diagnosis of parasitic meningitis, but enabled us to rule out other possible infections and conditions such as tuberculosis, aspergillosis, and malignancies. In addition, the neuroimaging of the patient showed multiple ring enhanced cystic lesions with eccentric high-density lesions within the cysts, suggestive of tapeworm scolices. The diagnosis of neurocysticercosis depends on neuroimaging, serologic tests, and histology. According to recent guidelines [16] and the UpToDate website [8], a definitive diagnosis of neurocysticercosis can be established by using the neuroimaging criteria (single or multiple active parenchymal cysts, with at least one cyst with a scolex on CT or MRS; resolution of cystic lesions after cysticidal drug therapy) and epidemiological exposure criteria, and the rapid clinical response to cysticidal therapy. Stool examination is insensitive in most patients with neurocysticercosis, as they have no viable intestinal tapeworms at the time of diagnosis [8], so the negative stool test result does not rule out the diagnosis of neurocysticercosis in our patient. Serologic tests should be conducted but are not diagnostic for neurocysticercosis [8]: Negative serologic results cannot exclude the diagnosis of neurocysticercosis, and positive results may reflect previous infection. Thus, the negative serologic tests of our patient did not exclude the diagnosis of neurocysticercosis, but helped us to exclude aspergillosus, cryptococcus, and tuberculosis. Neuroendoscopic brain biopsy is an accurate procedure for confirming neurocysticercosis, but it is usually not feasible in a critical care unit setting [17]. Without a brain biopsy, it was not possible to make a final diagnosis of the etiology of our patient’s encephalopathy. Fortunately, she was eventually cured.

### Pericardial effusion

The small pericardial effusion in our patient suggested the presence of pericarditis which might have been due to the systemic inflammatory response. During the pulmonary lobectomy, the thoracic surgeon did not find a substantial pericardial effusion and did not obtain pericardial tissue for a biopsy. Therefore, we could not confirm the etiology of her pericardial effusion. The etiology of pericardial diseases often remains unknown unless aggressive interventions are implemented [18].

### Pulmonary aspergillosis

Our patient developed foci of aspergillosis within a chronic lung abscess. The absence of microvascular fungal hyphae invasion, negative blood antigen test result, and negative blood culture for *Aspergillus* antigens suggest that our patient had chronic pulmonary aspergillosis but not invasive pulmonary aspergillosis [19]. Pulmonary aspergillomas are often found in the upper lobe of a pre-existing cavity, typically in a ball-like formation within the cavity, but may also be found in other locations within the lung if there is a pre-existing cyst or cavity. Our patient had a focus of aspergillosis within the cavity of the right middle lobe, but there was no obvious ball-like lesion within the lung abscess. She probably had *Aspergillus* colonization within the cavity caused by co-existing *Streptococcus intermedius* pneumonia, and she may not have needed antifungal therapy, especially once her cavity had been surgically removed. Considering her critical condition and mildly immunocompromised status, we decided to provide antifungal therapy for 2 weeks.

## Conclusion

A lowered level of consciousness is a common initial presentation in intensive care units and has many potential causes. Clinicians involved in critical care should be familiar with the symptoms and signs of all possible causes of a lowered level of consciousness for timely diagnosis, treatment, and better outcome. Multidisciplinary teamwork is especially important for such complex cases.

## Data Availability

All data generated or analyzed during this study are included in this published article.

## Abbreviations

CSF: Cerebrospinal fluid
CT: Computed tomography
CXR: Chest X-ray
DKA: Diabetic ketoacidosis
EEG: Electroencephalogram
HHS: Hyperosmolar hyperglycemic state
MRI: Magnetic resonance imaging
MRS: Magnetic resonance spectroscopy
